# Structural insights into the substrate transport mechanism of the amino acid transporter complex

**DOI:** 10.1016/j.jbc.2025.110569

**Published:** 2025-08-06

**Authors:** Haonan Yang, Tianhao Shi, Jing Dong, Ting Zhang, Yaning Li, Yingying Guo, Yafei Yuan, Liuqing Yang, Jintang Dong, Renhong Yan

**Affiliations:** 1 Department of Biochemistry, SUSTech Homeostatic Medicine Institute, School of Medicine, Key University Laboratory of Metabolism and Health of Guangdong, Institute for Biological Electron Microscopy, Southern University of Science and Technology, Shenzhen, Guangdong, China; 2 Department of Human Cell Biology and Genetics, School of Medicine, Southern University of Science and Technology, Shenzhen, Guangdong, China; 3 Beijing Frontier Research Center for Biological Structure, State Key Laboratory of Membrane Biology, Tsinghua-Peking Joint Center for Life Sciences, School of Life Sciences, Tsinghua University, Beijing, China

**Keywords:** LAT1, 4F2hc, cryo-EM, substrate transport, amino acid transporter, _L_-DOPA

## Abstract

The _L_-type amino acid transporter 1 (LAT1), in complex with its ancillary protein 4F2hc, mediates the sodium-independent antiport of large neutral amino acids across the plasma membrane. LAT1 preferentially transports substrates, such as _L_-leucine, _L_-tyrosine, and _L_-tryptophan, thyroid hormones, and drugs like 3,4-dihydroxyphenylalanine. Its pivotal role in cancer development and progression has established LAT1 as a promising therapeutic target. While prior studies have resolved the LAT1–4F2hc architecture and inhibitor interactions, the molecular basis of LAT1 substrate selectivity remains elusive. Here, we present the cryo-EM structures of LAT1–4F2hc bound to _L_-tyrosine, _L_-tryptophan, _L_-leucine, and 3,4-dihydroxyphenylalanine, revealing distinct substrate binding modes. Comparative structural analysis highlights differences between LAT1 and LAT2 in substrate coordination, driven by key residues near the binding pocket that influence transport efficiency. These findings advance our mechanistic understanding of the LAT1–4F2hc complex and provide valuable insights for structure-based drug design targeting LAT1.

Amino acids are indispensable nutrients essential for life, serving as precursors for a wide range of metabolites and as fundamental building blocks of proteins (1, 2, 3). Among the various amino acid transporters, the _L_-type amino acid transporter 1 (LAT1, SLC7A5), in complex with the heavy chain 4F2hc (CD98hc, SLC3A2), facilitates the transmembrane (TM) transport of neutral amino acids with midsized to large, less polar substrates, including _L_-leucine (Leu), _L_-isoleucine, _L_-valine, _L_-phenylalanine, _L_-tyrosine (Tyr), _L_-tryptophan (Trp), _L_-histidine, and _L_-methionine, in a sodium- and pH-independent manner (4, 5). In addition, LAT1–4F2hc mediates the transport of thyroid hormones, such as diiodothyronine (T2) and triiodothyronine (T3), as well as pharmaceutical compounds like 3,4-dihydroxyphenylalanine (_L_-DOPA) and gabapentin (6, 7, 8, 9, 10).

The LAT1–4F2hc complex is a representative member of the Heteromeric Amino Acid Transporter (HAT) family, composed of light and heavy subunits from the SLC7 and SLC3 families, respectively (11, 12, 13, 14, 15). LAT1 serves as the light chain, which is directly responsible for substrate transport, whereas 4F2hc acts as the heavy chain, ensuring proper localization of the complex on the plasma membrane and modulating transport activity (4, 5, 12, 16, 17). Dysregulation of LAT1–4F2hc is implicated in several pathological conditions, most notably cancer, where it promotes cell proliferation, angiogenesis, and metastasis (18, 19, 20). Elevated LAT1 expression levels are strongly associated with poor prognosis in various cancers (21, 22, 23, 24, 25, 26).

Beyond its oncogenic role, LAT1 is integral to the peripheral immune system, where it regulates immune cell activation *via* the mechanistic target of rapamycin 1 signaling pathway (27). Its expression is significantly upregulated during immune cell activation, and selective inhibition of LAT1 transport activity—such as with the specific inhibitor JPH203—impairs amino acid uptake and cytokine production in T cells (28, 29, 30). In addition, LAT1 mutations have been associated with neurological disorders and are pivotal for the transport of drugs like _L_-DOPA across the blood–brain barrier, facilitating its therapeutic efficacy in early-stage Parkinson’s disease (8, 31).

Structural studies have provided critical insights into the function of the LAT1–4F2hc complex (17, 32, 33, 34). The resolved overall structure of the complex exhibits extensive interactions among the extracellular domain, TM helices, and intracellular regions, with mutations in these regions significantly reducing transport activity (17). Earlier analyses identified the traditional substrate-binding pocket, with transport assays underscoring the importance of Gly255 in substrate selectivity (17, 32). Subsequent investigations of the LAT1–4F2hc complex bound to various inhibitors revealed a large hydrophobic cavity within the substrate-binding pocket, capable of accommodating inhibitors with bulky side chains (33, 34).

Like other solute carrier (SLC) transporters, LAT1–4F2hc operates *via* an alternating-access mechanism, cycling through distinct conformational states to facilitate substrate transport across the membrane (35). The gating residue Phe252 regulates transitions between inward-open, outward-facing occluded, and outward-open states by repositioning to control the substrate-binding site accessibility. In the inward-open conformation, the substrate-binding site is exposed to the cytoplasm, enabling substrate binding or release, as seen in the LAT1–4F2hc–2-amino-2-norbornanecarboxylic acid complex (17). Upon substrate binding, the transporter shifts to an occluded state, followed by extracellular-facing conformational changes where cytoplasmic helices constrict, closing the intracellular gate and trapping the substrate (outward-facing occluded) (33). Further opening of the extracellular gate transitions the transporter to the outward-open conformation, a critical checkpoint ensuring vectorial transport by facilitating substrate release into the extracellular space and exchange for another molecule (36).

Despite these advances, the detailed mechanisms underlying substrate recognition and transport by LAT1–4F2hc remain incompletely understood. In this study, we employ cryo-EM to examine the LAT1–4F2hc complex bound to various substrates, focusing on the role of key amino acid residues in its transport function.

## Results

### Structural determination of LAT1–4F2hc complex bound to different substrates

**Figure 1:**
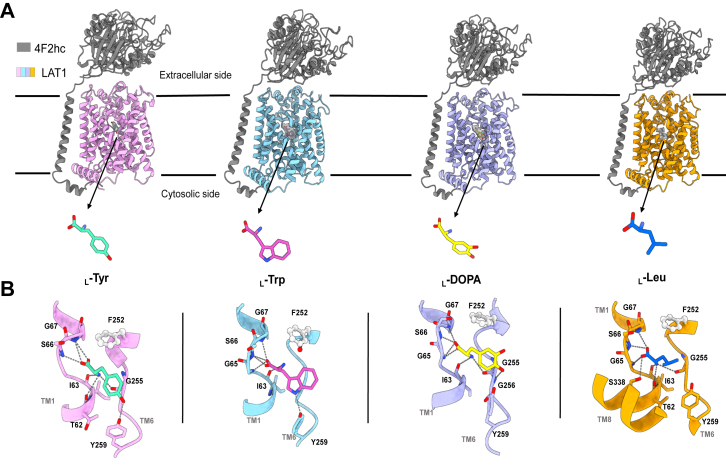
**Overall structure of the LAT1–4F2hc bound with four substrates.***A*, molecular structures of the four substrates on the *bottom*. LAT1 (colored *pink*, *sky blue*, *purple*, and *orange*) is shown in complex with four different substrates: _L_-Tyr (*green*), _L_-Trp (*hot pink*), _L_-DOPA (*yellow*), and _L_-Leu (*blue*). LAT1 is complexed with 4F2hc (*gray*), which is shown in all subunits for structural context. The cryo-EM density is wrapped around the substrate molecule in the form of a *transparent gray surface*. *B*, four substrate binding modes in LAT1. The substrate interaction sites are shown in different colors to correspond to the binding residues of _L_-Tyr (*pink*), _L_-Trp (*blue*), _L_-DOPA (*purple*), and _L_-Leu (*orange*). *Dashed lines* represent hydrogen bonds between the substrates and the surrounding residues, and key amino acids involved in hydrophobic interactions are indicated. _L_-type amino acid transporter 1.

To explore the substrate recognition and transport mechanism of LAT1–4F2hc complex, we determined the cryo-EM structures of the complex bound to various substrates: _L_-Tyr, _L_-Trp, _L_-Leu, and _L_-DOPA, at resolutions of 3.20 Å, 3.58 Å, 3.10 Å, and 3.56 Å, respectively (Fig. 1 and S1). Detailed cryo-EM sample preparation, data collection, processing, and model building procedures are provided in the "Experimental procedures" section (Figs. S1–S3 and Table S1). These structures are referred to as heteromeric amino acid transporter (HAT) + Tyr, HAT + Trp, HAT + Leu, and HAT + DOPA, respectively.

All four substrates bind to the conserved substrate-binding pocket of LAT1, located beneath the gating residue Phe252 (Fig. 1*B*). However, the binding of different substrates induces variations in the local binding patterns and conformational states. Specifically, the HAT + Tyr, HAT + Trp, and HAT + DOPA complexes adopt outward-facing occluded conformations, with Phe252 partially occluding the substrates. In contrast, the HAT + Leu complex exhibits a fully occluded conformation, with Phe252 completely covering the Leu substrate (Fig. 1*B*). The substrate-specific mechanisms underlying LAT1–4F2hc transport will be discussed in detail later. The electron density maps for four substrates are included in Figs. S2 and S3. Although the side chain of _L_-DOPA remains unclear at the current resolution, its orientation was modeled based on the available electron density and further supported by known interaction patterns observed in related structures.

### Substrate binding pattern of LAT1

The intricate mechanism of substrate recognition by LAT1 is elucidated through our structural insights. All four substrates are enclosed by the unwound regions of TM helices 1 and 6 (Fig. 1). In the HAT + Tyr complex, the carboxyl group of Tyr forms hydrogen bonds with the backbone amide groups of Ser66 and Gly67, whereas the substrate’s amino group is stabilized by the backbone carbonyl groups of Thr62, Ile63, and Gly255. Notably, the phenolic hydroxyl group of Tyr259 also forms a hydrogen bond with the phenolic hydroxyl group of Tyr, contributing to the stabilization of the Tyr–substrate interaction. In the HAT + DOPA complex, the carboxyl oxygen atoms of _L_-DOPA are hydrogen bonded with the backbone amide groups of Gly65, Ser66, and Gly67, whereas the substrate’s amino group is stabilized by the backbone carbonyl group of Ile63 and Gly255. Interesting, the para hydroxyl group of _L_-DOPA can form hydrogen bonds with the α-amino group of Gly256 and away from Tyr259 compared with the Tyr-bound complex (Fig. 1*B*). Given the current resolution, we refrain from overinterpreting the pattern specificity of _L_-DOPA binding and emphasize that our conclusions are based on the available data.

In the Trp-bound complex, the carboxyl oxygen atoms of Trp form hydrogen bonds with the backbone amide groups of Gly65, Ser66, and Gly67 in TM1. The α-amino group of Trp interacts with the backbone carbonyl group of Ile63. The side chain of Trp can also form hydrogen bonds with the hydroxyl of Tyr259. In the Leu-bound complex, the carboxyl oxygen atoms of Leu are hydrogen bonded with the backbone amine groups of Gly65, Ser66, and Gly67, and the carbonyl group of Ser338 in TM8, whereas the α-amino group is stabilized by the backbone carbonyl group of Thr62, Ile63, and Gly255. Notably, the hydrophobic side chain of Leu is engaged by the hydrophobic side chain of Phe252. A structural comparison reveals that Leu is positioned closer to the unwound region of TM1 compared with other substrates, potentially contributing to the occluded conformation (Fig. S4).

### Comparison of substrate binding in LAT1

**Figure 2:**
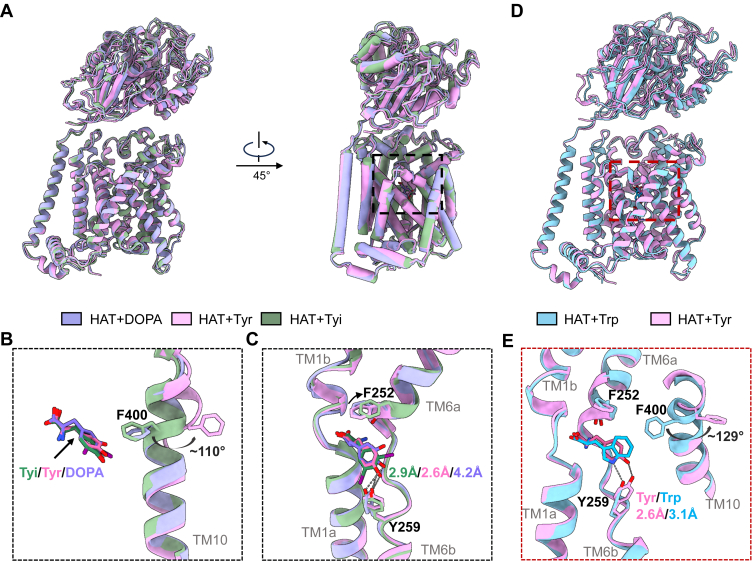
**Comparison of LAT1–4F2hc structures bound with different substrates.***A*, *cartoon* alignment model showing the structures of LAT1–4F2hc bound with L-DOPA (*purple*), Tyr (*pink*), and Tyi (*green*). *B*, the conformational shift of residue F400 in LAT1 when bound with Tyi, Tyr, and _L_-DOPA, showing a rotation of approximately 110°. *C*, detailed view of the shift in key residues, particularly F252 and Y259, in LAT1 bound with _L_-DOPA, Tyr, and Tyi, with distances of 2.9 Å, 2.6 Å, and 4.2 Å highlighted between the bound substrates and the residues. *D*, *cartoon* alignment model of LAT1–4F2hc structures bound with Trp (*blue*) and Tyr (*pink*). *E*, the conformational differences in key residues (F252, F400, and Y259) when LAT1 is bound with Tyr and Trp, with rotations of approximately 129° and distances of 2.6 Å and 3.1 Å between the residues and the substrates. _L_-type amino acid transporter 1.

We then analyzed the structure of LAT1–4F2hc complex bound to Tyr and compared it with the structure of LAT1–4F2hc bound with Diiodo-_L_-Tyrosine (HAT + Tyi) (Fig. 2, *A*–*C*). Notable differences were observed in key amino acids, including a significant shift in Phe252, known as the gating residue. Comparing with the Diiodo-_L_-Tyrosine-bound conformation, the Tyr-bound conformation exhibited a trend toward a reduced opening to the extracellular space. In addition, the distance between Tyr259 and the substrate underwent a distinct change (Fig. 2*C*). In the _L_-DOPA and Diiodo-_L_-Tyrosine-bound structures, Phe400 is oriented toward the binding pocket. In contrast, in the Tyr-bound conformation, Phe400 undergoes a substantial displacement of approximately 110°, accompanied by a significant reorganization of the local protein backbone. Similarly, in the comparison between the Tyr- and Trp-bound conformations, Phe400 shifts by approximately 129°, indicating a broader conformational rearrangement (Fig. 2, *B* and *E*).

To further understand these changes, we analyzed the LAT1–4F2hc complex bound to other substrates, including _L_-DOPA and Trp (Fig. 2). Comprehensive comparisons revealed that while these structures all presented outward-facing occluded conformations, subtle shifts were observed in the TM region, particularly in TM1 and TM6. In structure alignment of the Tyr, _L_-DOPA, and Diiodo-_L_-Tyrosine-bound conformation, the movement of Phe252 suggested a further opening toward the extracellular space in HAT + Tyi structure (Fig. 2*C*). The distance between the hydroxyl group of Tyr259 and the hydroxyl group of the substrate also underwent noticeable changes, with distances of 2.6 Å, 2.9 Å, 3.1 Å, and 4.2 Å for Tyr, Diiodo-_L_-Tyrosine, Trp, and _L_-DOPA, respectively (Fig. 2, *C* and *E*). Apart from the polar interaction between the substrate and Tyr259, other amino acids near the LAT1 pocket also influenced this distance, such as the interaction between the substrate and Phe400. In these structures, we observed a notable rearrangement of Phe400, suggesting that the interaction between the substrate and Phe400, such as halogen bonds, cation–π, and π–π interactions, corresponding to Diiodo-_L_-Tyrosine, _L_-DOPA, and Trp, respectively, could be the cause of this deviation. Further comparison with other resolved LAT1 conformations revealed that the orientation of Phe400 is not fixed (Fig. S5), suggesting that its position is part of a broader, concerted conformational rearrangement rather than being solely determined by local interactions. This dynamic repositioning may reflect states of full transport trajectory, rather than distinct substrate-specific stable states. Such flexibility likely plays a role in modulating substrate translocation through LAT1.

### Structural comparison between LAT1 and LAT2

**Figure 3:**
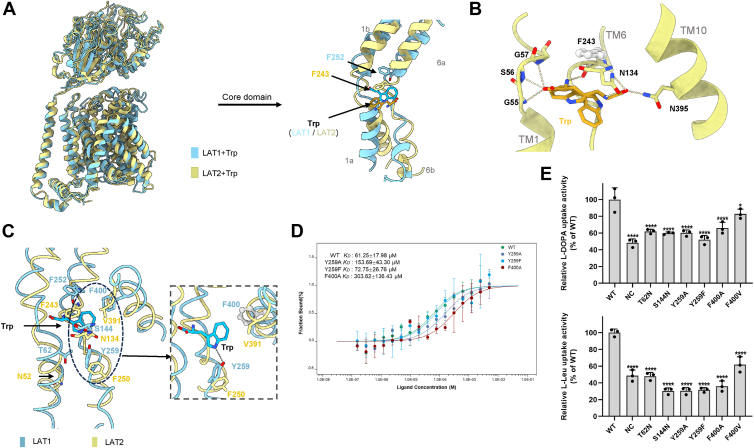
**Comparative analysis of LAT1 and LAT2 bound to Trp.***A*, overall structural alignment of LAT1 (*blue*) and LAT2 (*yellow*, PDB ID: 7CMH) bound with Trp, highlighting key differences in the core domain, particularly around Phe243, Phe252, and Tyr259. *B*, detailed view of Trp binding mode in LAT2, showing interactions with critical residues, such as Phe, Asn134, and Asn395 within the substrate binding pocket. *C*, comparison of the substrate binding sites in LAT1 and LAT2, focusing on the interactions of Trp with critical residues, including Phe252, Tyr259, and Phe400 in LAT1 (*blue*), and their corresponding counterparts in LAT2 (*yellow*). *D*, binding affinity measurements for WT and mutant LAT1 proteins with Trp, illustrating the reduced affinity of specific LAT1 mutations (Y259A, F400A, and Y259F) compared with the WT. KD values are indicated for each mutant. Data are presented as mean ± SD (n = 3). *E*, relative uptake activity of _L_-DOPA (*top*) and _L_-leucine (*bottom*) in WT and mutant LAT1 proteins, based on mass spectrometry results. The results indicate the impact of specific mutations on substrate transport efficiency, with significant differences observed for certain mutants, particularly T62N, S144N, Y259A, Y259F, and F400A. Data are shown as mean ± SD (n = 3). Statistical analysis was performed using Tukey’s multiple comparison test. Significant differences are indicated as follows: *p* < 0.05 (∗), *p* < 0.01 (∗∗), *p* < 0.001 (∗∗∗), and *p* < 0.0001 (∗∗∗∗). _L_-type amino acid transporter 1; PDB, Protein Data Bank.

The structures of LAT2 bound to Trp and Leu have also been solved (37). While LAT2 shares high sequence similarity with LAT1, comparison of their electron microscopy structures bound to the same substrate revealed a notable difference in the orientation of the substrate-binding pocket. Specifically, LAT2 bound to Trp adopts an inward-open conformation, whereas LAT1 bound with Trp exhibits an outward-facing occluded conformation (Fig. 3*A*). The discrepancy is likely attributed to differences in how the substrate interacts with the amino acids in pocket. In the first mode, the substrate’s carboxyl group forms hydrogen bonds with the backbone amino group of Gly55, Ser56, and Gly57 (TM1), whereas the amino group forms a hydrogen bond with the carboxyl group of Phe243 (TM6). In the second mode, the α-amino and α-carboxyl groups of Trp interact with the side chains of Asn134 (TM3) and Asn395 (TM10), forming additional hydrogen bonds (Fig. 3*B*). A comparison of the sequences and structures of LAT1 and LAT2 identified four key differing residues in the LAT1 pocket: Thr62, Tyr259, Ser144, and Phe400 (Figs. 3*C* and S7). In LAT1, the hydroxyl group of Tyr259 forms hydrogen bonds with the side chain of Trp, whereas Phe400 may engage in π–π interactions with the side chain of Trp. This binding mode differs from that observed in LAT2 (Fig. 3*C*).

To confirm the role of these residues in substrate transport, we analyzed key interaction sites identified in various LAT1 and LAT2 complexes. Thr62 serves as an interaction site in the LAT1–Leu and LAT1–Tyr complex, Ser144 in LAT1 does not participate in substrate binding in the LAT1–Trp complex, whereas its counterpart Asn134 in LAT2 appears to engage directly with Trp in a specific conformational state. Tyr259 is involved in substrate binding in both LAT1–Tyr and LAT1–Trp complexes. In addition, Phe400, located in TM10, may participate in the transport process, as this region undergoes structural rearrangement in the LAT1–Tyr complex. These variants were then assessed using a cell-based transport assay. All the mutants showed a notable reduction in the transport of _L_-DOPA or Leu, except for the F400V that exhibits a lower but not significant transport efficiency for the _L_-DOPA substrate, suggesting the critical role of these residues in the substrate transport (Fig. 3*E*). Interestingly, although Tyr259 does not directly interact with the _L_-DOPA or Leu substrates, its mutation may affect transport by perturbing local interaction networks or altering the conformational dynamics of the transporter during the substrate translocation cycle. Besides, microscale thermophoresis (MST) assays on LAT1 bound to Trp corroborated these findings, revealing that mutations at F400A and Y259A reduced the affinity of LAT1 for Trp by more than twofold (Fig. 3*D*). These findings suggest that Phe400 and Tyr259 could be critical residues for selective drug design with LAT1 and LAT2.

We also compared the structure of LAT1 and LAT2 bound with Leu. Similar to Trp binding pattern, the facing orientations of the complexes differ. Leu interacts primarily with unwound regions of TM1 and TM6 *via* hydrogen bonds to Ile63, Gly65, Ser66, Gly67, and Gly255, stabilizing its position in the binding pocket. A further hydrogen bond between Ser338 and Leu’s carboxyl group enhances binding affinity. Sequence alignment indicates that the majority of relevant residues involved in substrate interaction are conserved between LAT1 and LAT2, suggesting a shared substrate recognition mechanism despite their structural divergence (Fig. S6). Therefore, the distinct orientations of LAT1 and LAT2 for the same substrate may not only be influenced by the pocket environment but also by other regions of the protein complex.

## Discussion

**Figure 4:**
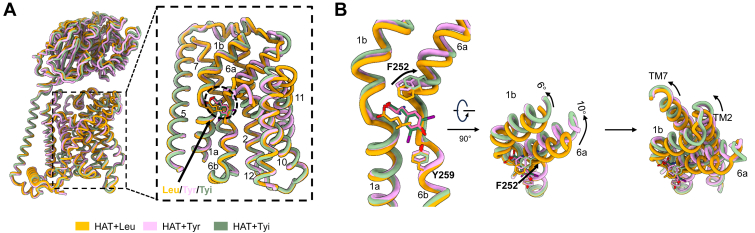
**Dynamic conformational changes in LAT1-mediated substrate transport.***A*, *cartoon* alignment model of LAT1–4F2hc structures bound with Leu (*yellow*), Tyr (*pink*), and Tyi (*green*). *B*, comparison of the LAT1 core region bound with different substrates, showing the interaction of key residues Phe252 and Tyr259 with the substrates and the movement tendency of the gate residue F252. The structures of TM1a and TM6b remain stable with no shift, whereas TM1b and TM6a are better illustrated through a 90° rotation, with rotations of 6° and 10°, respectively. The surrounding helices, TM2 and TM7, exhibit further outward rotation. _L_-type amino acid transporter 1.

The cryo-EM structures of LAT1–4F2hc complex bound to various substrates reveal a trend toward outward-facing conformations. To simulate the dynamic changes involved in LAT1-mediated substrate transport, we used typical structures bound to Leu, Tyr, and Diiodo-_L_-Tyrosine as examples (Fig. 4*A*). LAT1 bound to Leu adopts a fully occluded conformation, whereas LAT1 bound to Tyr and Diiodo-_L_-Tyrosine represent intermediate states, transitioning from outward-facing occluded to outward-open conformations (Fig. 4*A*). Notably, TM1 and TM6 exhibit notable swinging motions, with the gating residue Phe252 progressively moving away from the center of the substrate-binding site. Further analysis reveals that TM1a and TM6b do not undergo substantial changes, whereas TM1b and TM6a rotate outward by about 6° and 10°, respectively. The surrounding helices also experience shifts, with TM2 and TM7 swing outward, creating additional space for substrate release (Fig. 4*B*).

Interestingly, LAT1 and LAT2 exhibit different facing orientations when bound to the same substrates (Trp or Leu). While the residues involved in Leu coordination are conserved between the two transporters, those coordinating Trp differ. This discrepancy may arise because substrate binding conformation is influenced not only by the pocket environment but also by the other regions of the protein complex. Alternatively, structural variations could result from differences in protein particle states during cryogenic sample preparation.

**Figure 5:**
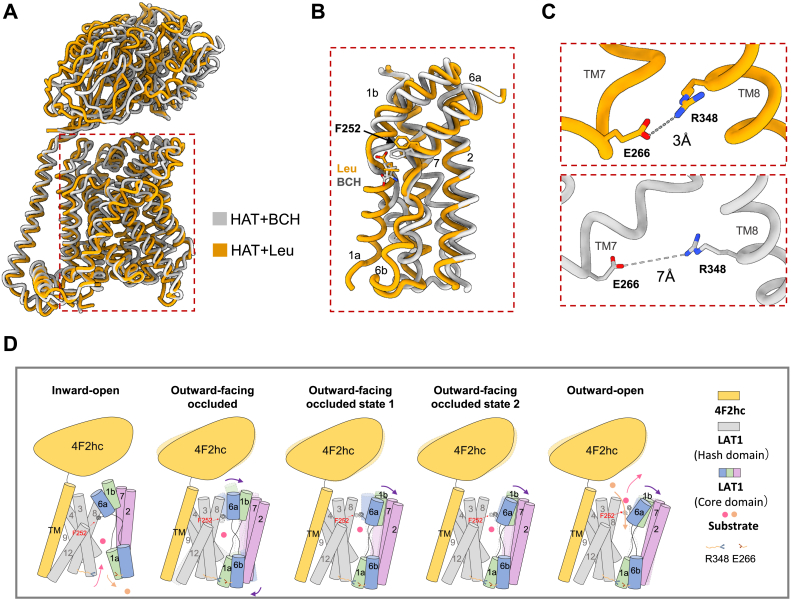
**Putative working model of LAT1–4F2hc complex.***A*, structural alignment of the LAT1–4F2hc complex bound with BCH (*light gray*) and leucine (*orange*), highlighting the conformational differences induced by the binding of these substrates. *B*, detailed conformational changes of the LAT1–4F2hc complex upon binding with BCH and leucine. The helices TM1a and TM6b show that LAT1 adopts an inward-open conformation when bound to BCH and an outward-open conformation when bound to leucine. *C*, interaction between residues Glu266 and Arg348 in different conformational states, showing a distance change from 7 Å to 3 Å, representing transitions during the transport cycle. *D*, proposed working model of the LAT1–4F2hc transport cycle, illustrating the transitions from inward-open to outward-open states. LAT1–BCH complex (PDB ID: 8KDH) represents the inward-open conformation. LAT1–Leu complex (PDB ID: 8X0W) represents an outward-facing occluded conformation. LAT1–Tyr complex (PDB ID: 8IDA) and LAT1–Tyi complex (PDB ID: 7DSQ) capture outward-facing occluded states (−1 and −2). y^+^LAT1–Arg complex (PDB ID: 8YLP) shows an outward-open state. This highlights the key steps involved in substrate translocation across the membrane. _L_-type amino acid transporter 1; PDB, Protein Data Bank; BCH, 2-amino-2-norbornanecarboxylic acid.

Based on previous and current structural findings, including the inward-open state, we propose a more comprehensive model for LAT1 substrate binding and transport (Fig. 5) (17, 33, 38). Our analysis suggests that LAT1 initially accepts substrate binding in the inward-open state. Subsequently, motions in TM1, TM2, TM6, TM7, and TM8 cause the lower gate to close, reducing the spatial distance between Glu266 and Arg348 from 7 Å to 3 Å (Fig. 5*C*). This is followed by continued shifts in TM1b and TM6a, gradually opening the upper gating residue—Phe252, transitioning the protein from outward-facing occluded intermediate states to outward-open conformation. Finally, the gate fully opens, allowing new external substrates to enter, thereby completing the substrate transport cycle.

Notably, key residues near the LAT1 substrate-binding pocket are crucial for substrate recognition, stabilization, and transport. Phe252 serves as a gate to prevent substrate leakage, whereas Tyr259 interacts with different substrates to facilitate recognition and stabilization. Phe400, through its dynamic flexibility, may influence the transport efficiency of different substrates. These interactions contribute to variations in substrate recognition and transport efficiency. Substrates with large side chains generally exhibit lower transport rates but higher affinity for LAT1 (39). Substrates with low transport rates also have the potential to serve as scaffolds for developing high-affinity inhibitors, providing a valuable direction for drug scaffold selection in future drug design.

In sum, this study employs high-resolution cryo-EM to analyze the structures of LAT1–4F2hc complex bound to different substrates, providing new insights into the substrate recognition and transport mechanisms of LAT1–4F2hc complex. Our findings highlight that key residues in the binding pocket primarily govern substrate recognition, whereas surrounding residues contribute to the remodeling of TMs, collectively facilitating substrate transport. These discoveries not only expand our understanding of LAT1 function but also provide a crucial structural foundation for drug development.

## Experimental procedures

### Protein expression and purification

The full-length human complementary DNA of LAT1 (accession number: NM_003486.7) was cloned into pCAG vector with N-terminal FLAG, and 4F2hc (isoform b, accession number: NM_001012662.2) was cloned into pCAG vector with N-terminal 10∗HIS tag.

For protein expression, LAT1 and 4F2hc were coexpressed in human embryonic kidney 293F (HEK293F) cells (Invitrogen), which were cultured in SMM 293-TII-N medium (Sino Biological, Inc) at 37 °C under 5% CO_2_ in a Multitron-Pro shaker (Infors, 130 rpm). To achieve coexpression of LAT1 and 4F2hc, 3 mg of polyethylenimines (YEASEN), 0.75 mg of the LAT1 plasmid, and 0.75 mg of the 4F2hc plasmid were preincubated with 50 ml fresh medium for 15 min and added into cell culture whose cell density reached 2.0 × 10^6^/ml. Sixty hours after transfection, cells were harvested by centrifugation at 3500*g* for 15 min and resuspended in a buffer containing 25 mM Hepes (pH 7.5), 150 mM NaCl, and mixture of three protease inhibitors, aprotinin (1.3 μg/ml, AMRESCO), pepstatin (0.7 μg/ml, AMRESCO), and leupeptin (5 μg/ml, AMRESCO).

For protein purification, after incubating with 1% (w/v) lauryl maltose neopentyl glycol (Anatrace) supplemented with 0.1% (w/v) cholesteryl hemisuccinate Tris salt (Anatrace) at 4 °C for 2 h, cells were centrifugated at 14,000*g* for 50 min to remove the cell debris. The supernatant was loaded onto anti-FLAG G1 affinity resin (Genscript). The resin was washed with the wash buffer containing 25 mM Hepes (pH 7.5), 150 mM NaCl, 0.01% (w/v) glyco-diosgenin (GDN; Anatrace), following by protein eluted with wash buffer plus 0.2 mg/ml FLAG peptide. Then elution of anti-FLAG G1 affinity resin was further purified with nickel–nitrilotriacetic acid affinity resin (Qiagen). Wash buffer and elution buffer of nickel resin was the wash buffer mentioned above plus 10 mM and 300 mM imidazole, respectively. Then the protein complex was subjected to size-exclusion chromatography (Superose 6 Increase 10/300 GL; GE Healthcare) in buffer containing 25 mM Hepes (pH 7.5), 150 mM NaCl, and 0.01% GDN. The peak fractions were collected and concentrated for EM analysis.

### *In vitro* transport activity assay

The construction of gene mutants was achieved through a two-step PCR method, building upon the original WT plasmid. The following primers were used (only four forward primers are shown, inverted sequence for reverse primer):

Thr62Asn: GCCATCATCGTGGGGAACATTATCGGCTCG

Ser144Asn: CATCCGGCCTTCAAACCAGTACATCGTGGCCCTGGTC

Tyr259Phe: GCCTATGGAGGATGGAATTTCTTGAATTTCGTCACAGAGG

Tyr259Ala: GCCTATGGAGGATGGAATGCCTTGAATTTCGTCACAGAGG

Phe400Val: CTCCGTCATCAACTTCGTCAGCTTCTTCAACTGGCTCTGC

Phe400Ala: CTTCTCCGTCATCAACTTCGCCAGCTTCTTCAACTGGCTCTGC

For _L_-DOPA uptake assays, firstly, HEK293 cells were seeded into 6-well plates and transfected with LAT1-WT and mutants in conjunction with 4F2hc for 48 h. Following the aspiration of the culture medium, the cell monolayers were preincubated for 10 min in Hanks’ medium (Beyotime) at a temperature of 37 °C. The cells were then incubated with 250 μM _L_-DOPA (Sigma, 98% purity) for 20 min in 1.5 ml Hanks’ medium. Subsequently, the cells were washed twice with ice-cold PBS and harvested in 0.3 ml of 10 mM perchloric acid containing 5 μg/ml methyldopa (TargetMol, 99%), which is used as an internal standard solution. Using a Q Exactive mass spectrometer coupled with an UltiMate 3000 liquid chromatography system (ThermoFisher), 10 μl supernatant was injected into an RPLC column (2.1 × 100 mm, 1.9 μm; Thermo Scientific) and ran through the column at a flow rate of 0.3 ml/min, along with data acquisition. Mobile phase A consisted of 0.1% formic acid in H_2_O, whereas mobile phase B consisted of 0.1% formic acid in acetonitrile. The mass spectrometer was operated in positive mode with a spray voltage of 3.5 kV. Full scan mass spectrometry data were recorded from the *m/z* range of 80 to 600.

For leucine uptake assays, HEK293T cells were seeded into 6-well plates and transfected with the pCAG-FLAG-LAT1 and its mutants in the presence of 4F2hc plasmid. After 48 h of transfection, cells were cultured in a medium containing 2 mM leucine-^13^C^15^N (Cambridge Isotope Laboratories) for 12 h. Subsequently, cells were washed three times with ice-cold PBS and harvested in 80% methanol containing 0.04 μM L-4-chlorophenylalanine (Sigma–Aldrich). Cells were broken by repeated freezing in liquid nitrogen and thawed at room temperature, and the supernatant was collected by centrifugation at 12,000 rpm for 10 min at 4 °C. Mass spectrometry analysis data were obtained under the same conditions.

### MST assay

LAT1 (WT, F400A, Y259A, and Y259F) were cloned into a pCAG vector with GFP and N-terminal FLAG tag and overexpressed in HEK293F cells together with 4F2hc. The purified proteins were diluted to 400 nM in dilute buffer (25 mM Hepes [pH 7.5], 150 mM NaCl, and 0.01% GDN). A tryptophan solution (30 mM) was prepared in the same buffer and serially diluted into 16 concentrations. Protein–ligand mixtures were incubated at 4 °C for 10 min before analysis using the Monolith NT.115 instrument (NanoTemper Technologies) at medium MST power. Data from three independently performed experiments were fitted to the single binding model *via* the MO Affinity analysis software version 2.3 (NanoTemper Technologies).

### Cryo-EM sample preparation and data acquisition

The purified LAT1–4F2hc complex was incubated with 10 mM Leu, 1 mM Trp, 1 mM _L_-DOPA, or 1 mM Tyr for 2 h and concentrated to ∼10 mg/ml. Aliquots (3.5 μl) of the mixture were placed on glow-discharged holey carbon grids (Quantifoil Au R1.2/1.3). The grids were blotted for 3 s or 3.5 s and flash-frozen in liquid ethane cooled by liquid nitrogen with Vitrobot (Mark IV; Thermo Fisher Scientific). The prepared grids were transferred to a Titan Krios operating at 300 kV equipped with Gatan K3 Summit detector and GIF Quantum energy filter. A total of 2878, 2276, 2609, and 2771 movie stacks were automatically collected using AutoEMation and EPU software (Thermo Fisher Scientific) for LAT1–4F2hc + Leu, LAT1–4F2hc + Trp, LAT1–4F2hc + _L_-DOPA, and LAT1–4F2hc + Tyr, respectively, with a slit width of 20 eV on the energy filter and a preset defocus range from −1.3 μm to −1.8 μm in super-resolution mode. The total electron dose was approximately 50 e−/Å2 for each stack, which contained 32 frames. The stacks were motion corrected and dose-weighted 2 with MotionCor2 and binned twofold, resulting in a pixel size of 0.855 Å/pixel, 1.095 Å/pixel, or 1.087 Å/pixel for the LAT1–4F2hc + Leu, LAT1–4F2hc + Trp, LAT1–4F2hc + _L_-DOPA, or LAT1–4F2hc + Tyr complex, respectively. The defocus values were estimated with Gctf (40).

### Data processing

The cryo-EM structures of LAT1–4F2hc were solved by cryoSPARC. Patch-based contrast transfer function (CTF) estimation was used to estimate the CTF correction parameters of micrographs in cryoSPARC. Particles were automatically picked using Blob picker and Template picker. Several rounds of 2D classification were performed, and the selected particles from 2D classification were subject to *ab initio* reconstruction and several cycles of heterogeneous refinement with C1 symmetry. Particles (LAT1–4F2hc + Leu, LAT1–4F2hc + Trp, and LAT1–4F2hc + _L_-DOPA) from the best class were subjected to nonuniform refinement, local CTF refinement, and local refinement. And particles (LAT1–4F2hc + Tyr) from the best classes were imported to Relion 3.0 (MRC Laboratory of Molecular Biology), which were subjected to a global angular searching 3D classification using the cryo-EM map of the LAT1–4F2hc + Tyr from cryoSPARC as the initial model with only one class. For each of the last several iterations of the global angular searching 3D classification, a local angular searching 3D classification was performed, during which the particles were classified into four classes. Nonredundant good particles were selected from the local angular searching 3D classification. Then, these selected particles were subjected to multireference 3D classification, local defocus correction, 3D autorefinement, and postprocessing. The resolution was estimated with the gold-standard Fourier shell correlation 0.143 criterion with high-resolution noise substitution. Refer to the Experimental procedures section of Figures S1, S3 and Table S1 for details of data collection and processing.

### Model building and structure refinement

The construction of the model was executed using Phenix (Berkeley Lab) and Coot (MRC Laboratory of Molecular Biology). Initially, the structure of the LAT1–4F2hc complex (Protein Data Bank [PDB] ID: 7DSQ) served as the starting model, which was subsequently aligned with the corresponding electron density maps utilizing ChimeraX, and subsequent refinement was carried out using real-space refinement in Phenix. Additional improvements were made through manual adjustments in Coot. Detailed refinement statistics is provided in Table S1.

## Data availability

Cryo-EM maps and molecular models have been deposited in the Electron Microsopy Data Bank (EMDB) and PDB, respectively. Accession codes are listed here. Atomic coordinates and EM density maps of LAT1–4F2hc complex bound with L-Tyr (PDB ID: 8IDA; EMDB ID: EMD-35361), _L_-DOPA-bound state (PDB ID: 8J8L; EMDB ID: EMD-36073), _L_-Trp-bound state (PDB ID: 8J8M; EMDB ID: EMD-36074), and L-Leu-bound state (PDB ID: 8X0W; EMDB ID: EMD-36074) have been deposited in the PDB and the EMDB, respectively. All other data will be made available upon request. Source data are provided with this article. Correspondence and requests for materials should be addressed to R. Y. (yanrh@sustech.edu.cn).

## Supporting information

This article contains supporting information.

## Conflict of interest

R.Y. is an investigator of SUSTech Institute for Biological Electron Microscopy. The authors declare that they have no conflicts of interest with the contents of this article.
