# Supplementary material for: Structural insights into the substrate transport mechanism of the amino acid transporter complex
Source: J Biol Chem. 2025 Aug 6;301(9):110569. doi: 10.1016/j.jbc.2025.110569 (PMC12444478; doi:10.1016/j.jbc.2025.110569)
Supplement: Supporting Figures and Tables [file mmc1.pdf]

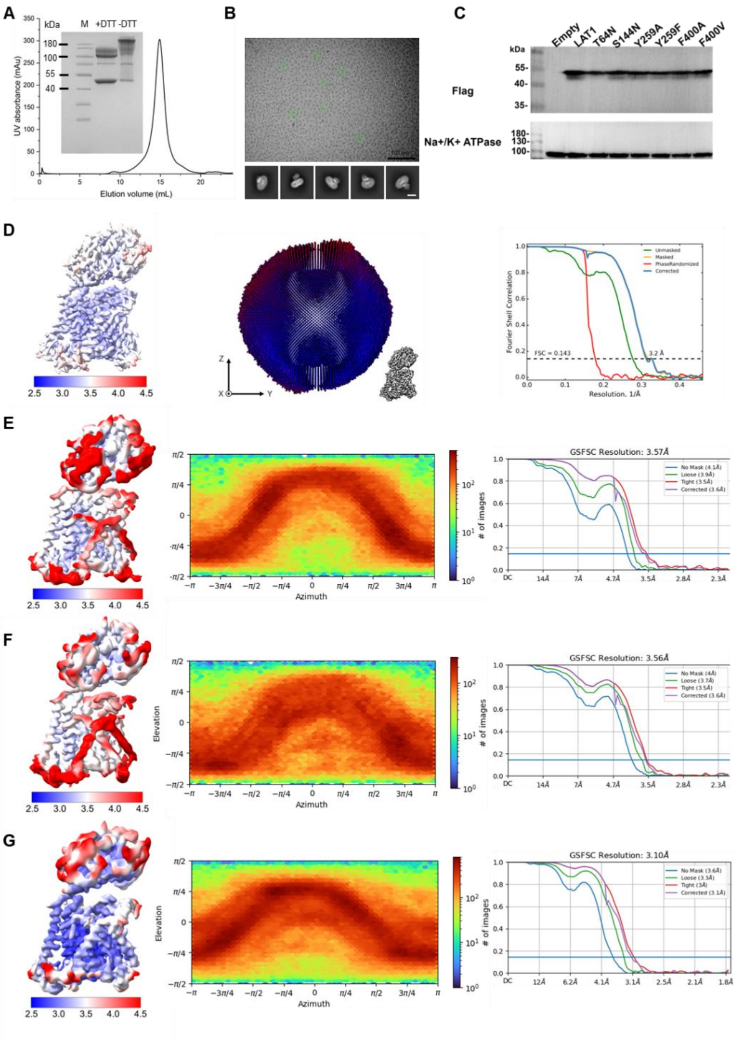

Figure S1. Cryo-EM analysis of LAT1-4F2hc complex

A Representative SEC purification of the LAT1-4F2hc complex. Inset, SDS–PAGE under reducing (+dithiothreitol (DTT)) or oxidizing (−DTT) conditions, visualized by Coomassie blue staining.

B Representative cryo-EM micrograph and 2D class averages of cryo-EM particle images. The scale bar in 2D class averages is 10 nm.

C Western blot analysis of wild-type and mutant LAT1 expression in HEK293T cells. Na^{+}/K^{+}-ATPase was used as a loading control.

D-G ***Left***: local resolution maps for the 3D EM reconstruction of the overall structure of the LAT1-4F2hc+Tyr, LAT1-4F2hc+Trp, LAT1-4F2hc+L-DOPA and LAT1-4F2hc+Leu; ***Middle***: Euler angle distribution of the complex; ***Right***: Gold standard FSC curve of the cryoSPARC 3D reconstruction of the whole map of the complex.

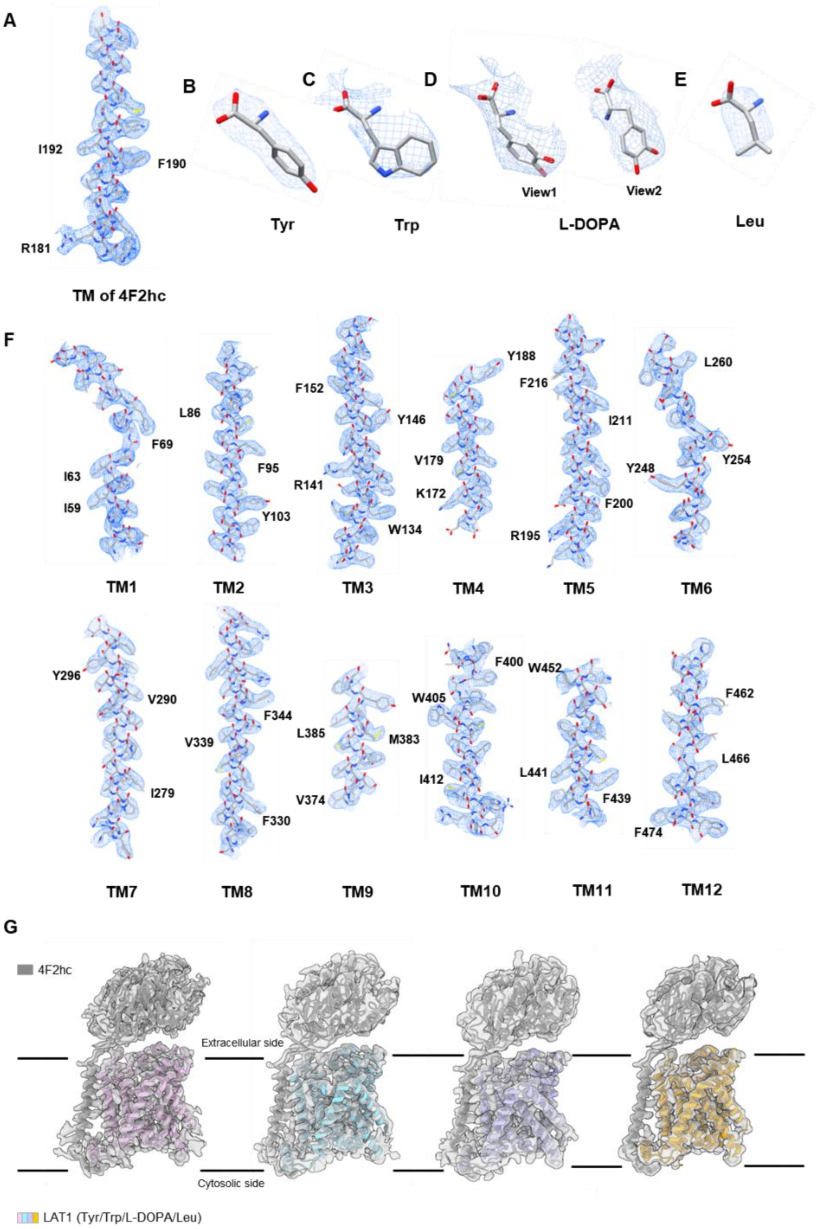

Figure S2. Cryo-EM analysis of LAT1-4F2hc complex

A Cryo-EM density map of transmembrane domain of 4F2hc is shown at threshold of 6 σ.

B-E Cryo-EM density maps of substrates of the LAT1-4F2hc+Tyr, LAT1-4F2hc+Trp, LAT1-4F2hc+L-DOPA, LAT1-4F2hc+Leu are shown at threshold of 8 σ.

F Cryo-EM density map of transmembrane domains of LAT1bound to Tyr substrate are shown at threshold of 6 σ.

G The architectures of LAT1-4F2hc complex bound to Tyr, Trp, L-DOPA, and Leu, respectively.

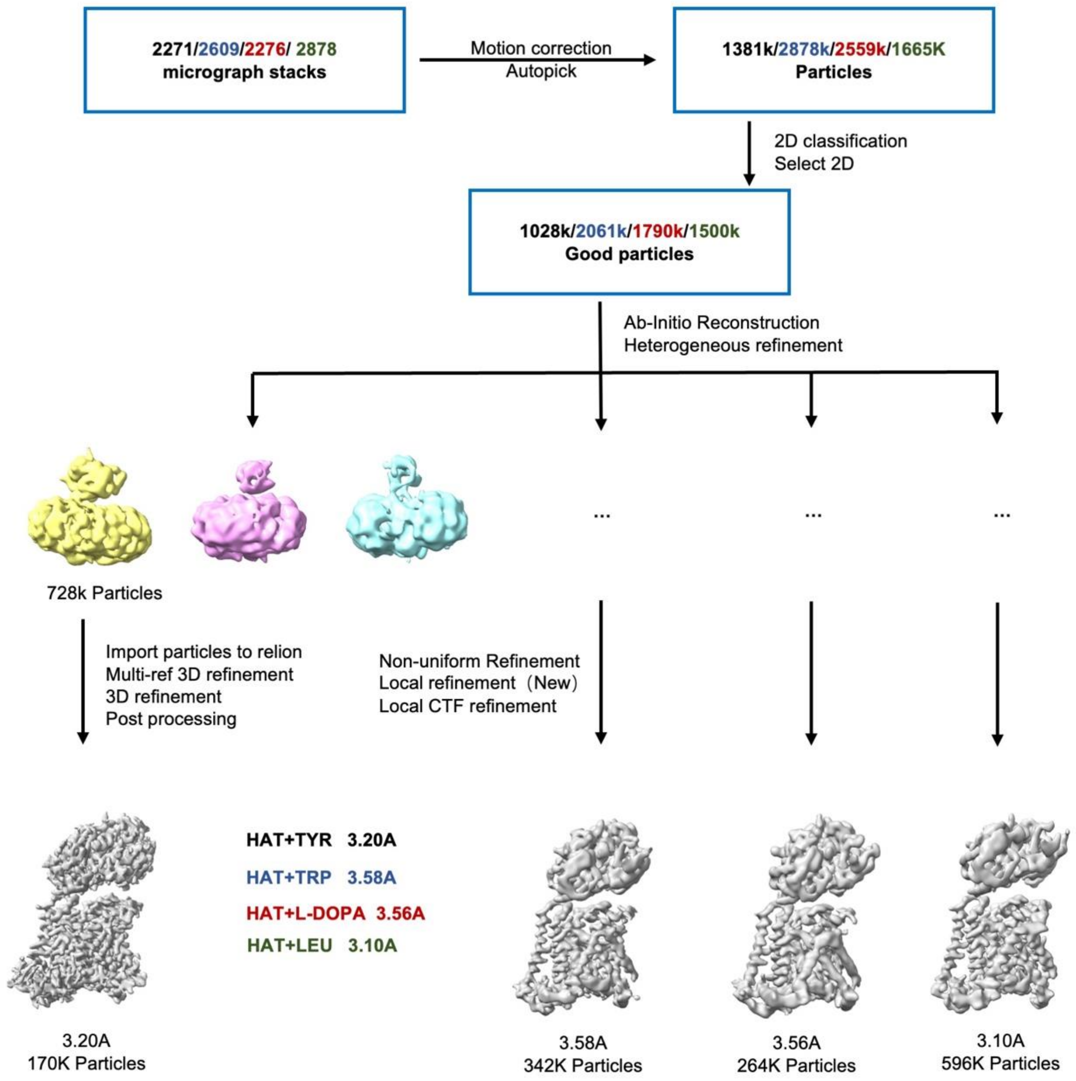

Figure S3. Cryo-EM data processing workflow for the LAT1-4F2hc complex

Flowchart for cryo-electron microscopy data processing. For detailed information, please refer to the 'Data Processing' section in the Methods.

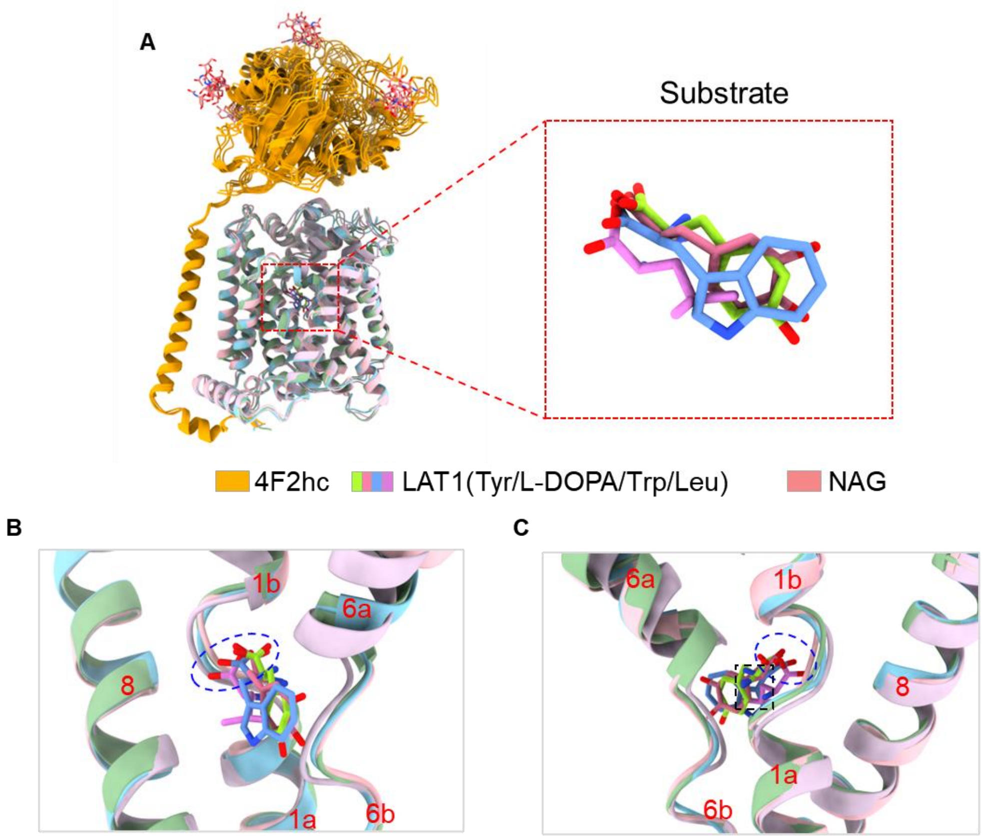

Figure S4. Differential substrate binding positions in the LAT1-4F2hc complex

A Structural Comparison of LAT1-4F2hc Binding to different Substrates. The red square highlights a magnified view of the substrate in the structure. The colored boxes correspond to detailed images in **B** and **C**.

B-C Different Perspectives of Substrate Position within the LAT1 Pocket. The blue circle indicates the C-terminus of the substrate, while the black square indicates the N-terminus of the substrate. Leucine occupies a position in the binding pocket that is closer to the wound region of TM1 compared to other substrates.

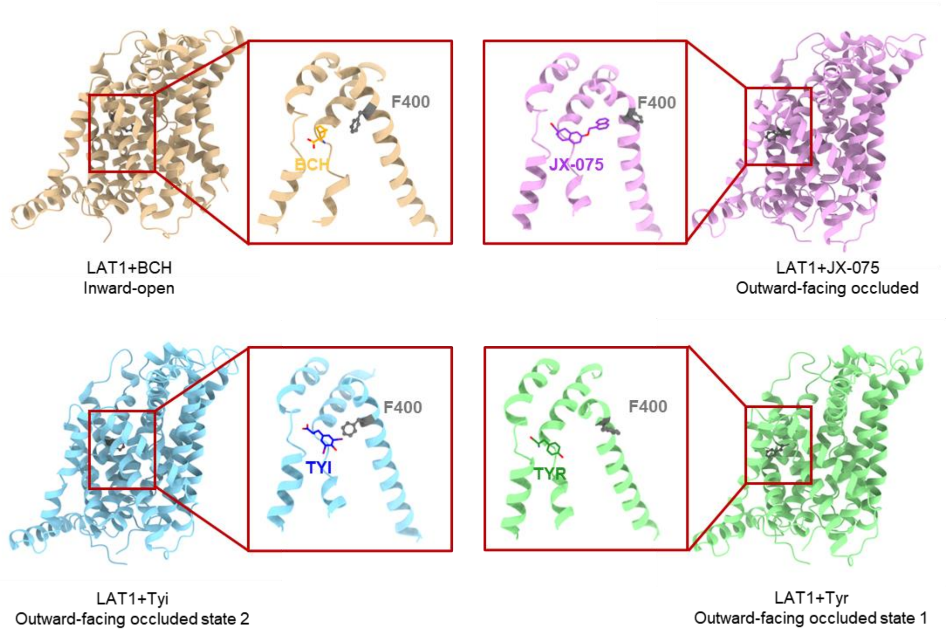

Figure S5. The position of F400 in different conformations of the LAT1-4F2hc

Displaying the structure of LAT1 only, the red square highlights the substrate and amino acid F400, with F400 emphasized in shades of black-gray. Structures of LAT1 in complex with BCH (PDB ID: 6irt), JX-075 (PDB ID: 7dsk), Diiodo-L-tyrosine (TYI) (PDB ID: 7dsq), Tyr are coloured in yellow, pink, blue and green, respectively.

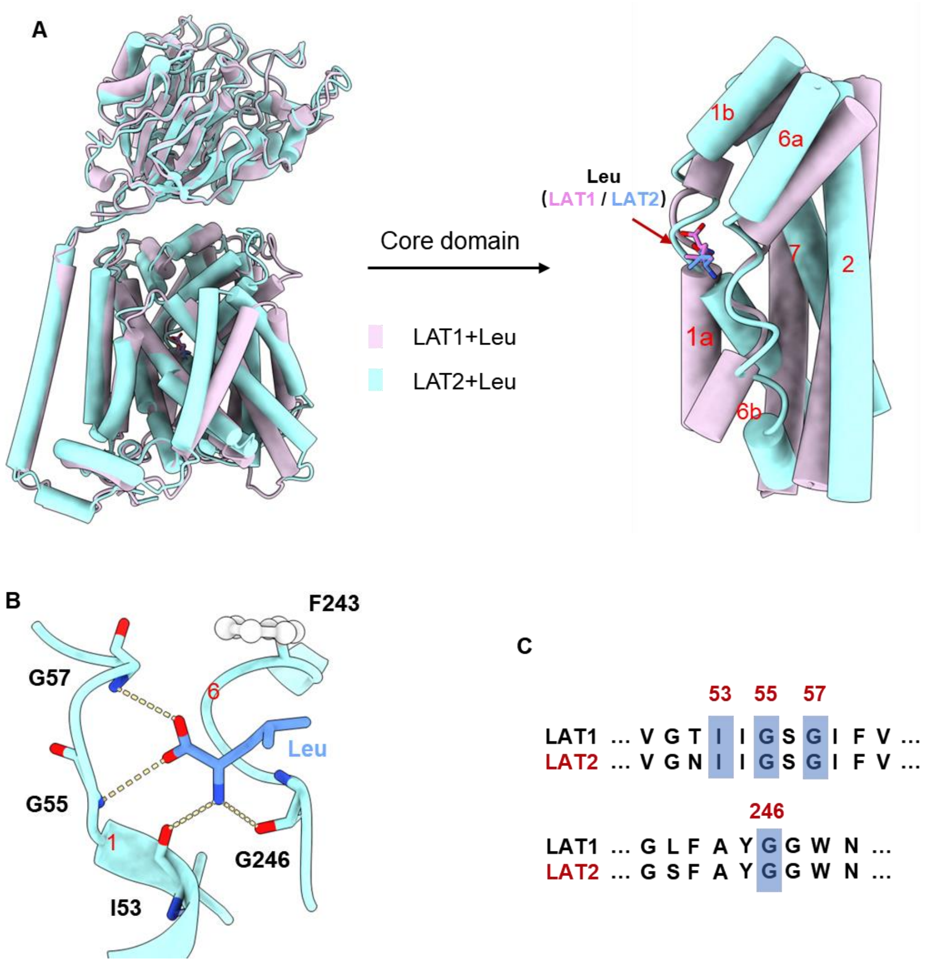

Figure S6. Conformational change between the LAT1-4F2hc and LAT2-4F2hc in complex with Leu

A Comparison of the LAT1-4F2hc and LAT2-4F2hc (PDB ID: 7cmi) in complex with Leu. The core domain of the LAT1 emphasizing the orientation of the pocket opening facing different directions.

B Leu binding mode in LAT2. The yellow dashed lines represent polar interactions. Phe243 (gating residue) is shown in sphere style.

C Local sequence alignment of LAT1 and LAT2. The amino acids in the pocket of LAT2 interacting with leucine are conserved in LAT1.

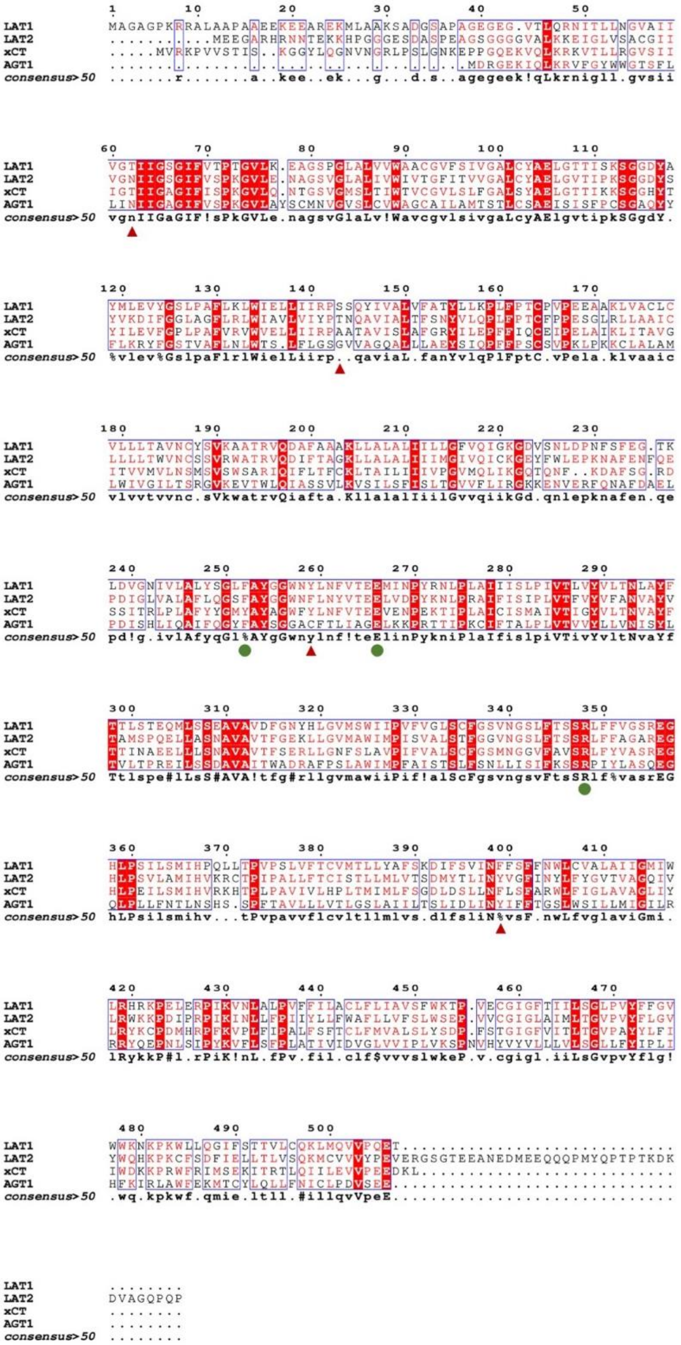

Figure S7. Sequence alignment of LAT1 homologues

The sequences are aligned using clustalW. The four aligned sequences are LAT1, LAT2, xCT and AGT1 from Homo sapiens. Residues that are identical or conserved in at least two sequences are colored in red, and the common conserved sequences are labeled with blue boxes. The red triangles denote mutated amino acids, while the green circles indicate gating amino acids.The UNIPROT IDs of aligned sequences are listed as below：

LAT1: Q01650; LAT2: Q9UHI5; xCT: Q9UPY5; AGT1: Q8TCU3.

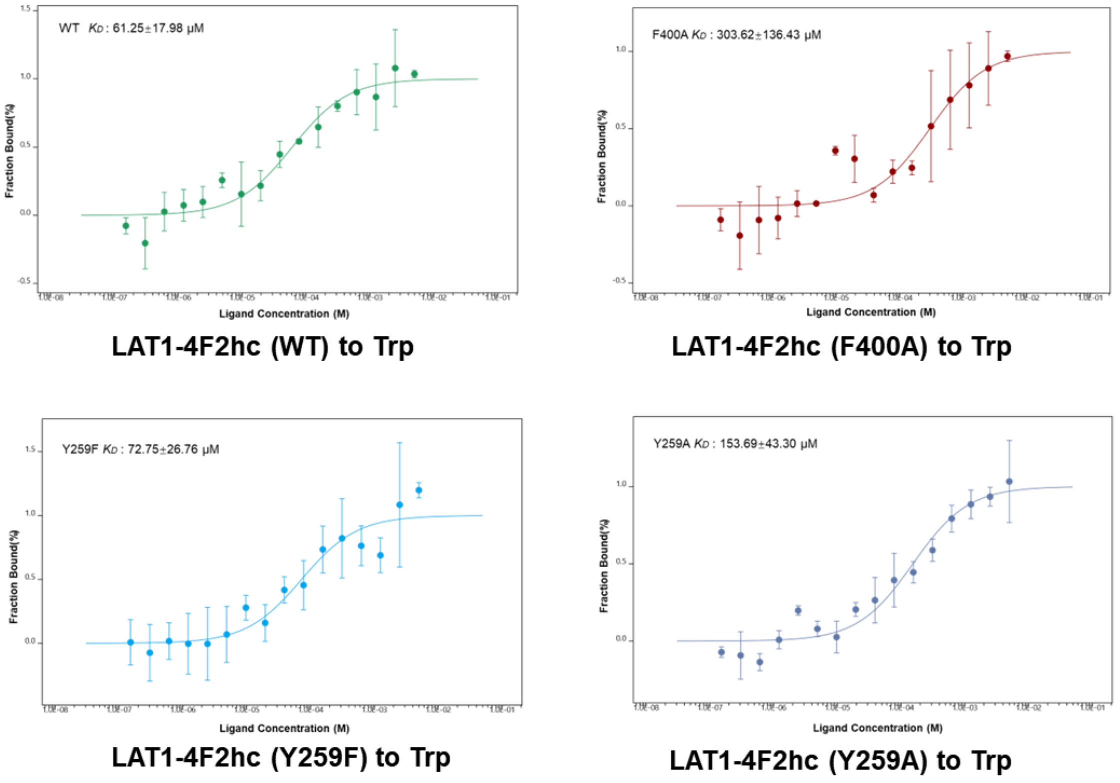

Figure S8. Additional information about Microscale thermophoresis (MST) assay MST assays of the substrate (Trp)-binding affinity of LAT1-4F2hc, respectively. Data from three independently performed experiments were fitted to the single binding model via the MO. Affinity analysis software version 2.3 (NanoTemper Technologies), error bars represent SD (standard deviation).

Table S1. Data collection, 3D reconstruction and model statistic

**Data collection**				
EM equipment	Titan Krios (Thermo Fisher Scientific)			
Voltage(kV)	300			
Detector	Gatan K3 Summit			
Energy filter	Gatan GIF Quantum, 20 eV slit			
sample	HAT+Tyr	HAT+Trp	HAT+L-DOPA	HAT+Leu
Pixel size(Å)	1.087	1.095	1.095	0.855
Electron dose(e-/Å2)	50			
Defocus range(μm)	-1.3 ~ -1.8			
Number of collected micrographs	2771	2609	2276	2878
**3D Reconstruction**				
Software	Relion 3.0 & cryoSPARC			
Number of used particles (Overall)	170,888	342,075	263,809	596,542
Resolution(Å)	3.20	3.57	3.56	3.10
Symmetry	C1			
Map sharpening B-factor (Å2)	-90.0	-159.0	-176.3	-153.0
**Refinement**				
Software	Phenix			
Cell dimensions				
a=b=c(Å)	278.272	280.320	280.320	246.24
α=β=γ(°)	90	90	90	90
Model composition				
Protein residues	934	932	932	934
Side chains assigned	934	932	932	934
Sugar	4	4	4	4
Lipid	1	1	1	1

ligand	1	1	1	1
R.m.s deviations				
Bonds length(Å)	0.003	0.003	0.003	0.002
Bond Angle(°)	0.592	0.657	0.653	0.571
Ramachandran plot statistics(%)				
Preferred	95.05	95.26	95.47	94.84
Allowed	4.95	4.74	4.53	5.16
Otlier	0	0	0	0
